# Impact of an Intervention to Use a Measles, Rubella, and Polio Mass Vaccination Campaign to Strengthen Routine Immunization Services in Nepal

**DOI:** 10.1093/infdis/jix164

**Published:** 2017-07-01

**Authors:** Aaron S. Wallace, Rajendra Bohara, Steven Stewart, Giri Subedi, Abhijeet Anand, Eleanor Burnett, Jagat Giri, Jagat Shrestha, Suraj Gurau, Sameer Dixit, Rajesh Rajbhandari, W. William Schluter

**Affiliations:** 1 Global Immunization Division, Centers for Disease Control and Prevention, Atlanta, Georgia; 2 World Health Organization Nepal, Kathamandu, Nepal; 3 Ministry of Health, Kathamandu, Nepal; 4 Center for Molecular Development Network, Kathamandu, Nepal

**Keywords:** polio, measles, supplementary immunization activities, immunization systems

## Abstract

**Background:**

The potential to strengthen routine immunization (RI) services through supplementary immunization activities (SIAs) is an important benefit of global measles and rubella elimination and polio eradication strategies. However, little evidence exists on how best to use SIAs to strengthen RI. As part the 2012 Nepal measles-rubella and polio SIA, we developed an intervention package designed to improve RI processes and evaluated its effect on specific RI process measures.

**Methods:**

The intervention package was incorporated into existing SIA activities and materials to improve healthcare providers’ RI knowledge and practices throughout Nepal. In 1 region (Central Region) we surveyed the same 100 randomly selected health facilities before and after the SIA and evaluated the following RI process measures: vaccine safety, RI planning, RI service delivery, vaccine supply chain, and RI data recording practices. Data collection included observations of vaccination sessions, interviews with the primary healthcare provider who administered vaccines at each facility, and administrative record reviews. Pair-matched analytical methods were used to determine whether statistically significant changes in the selected RI process measures occurred over time.

**Results:**

After the SIA, significant positive changes were measured in healthcare provider knowledge of adverse events following immunization (11% increase), availability of RI microplans (+17%) and maps (+12%), and awareness of how long a reconstituted measles vial can be used before it must be discarded (+14%). For the SIA, 42% of providers created an SIA high-risk villages list, and >50% incorporated this information into RI outreach session site planning. Significant negative changes occurred in correct knowledge of measles vaccination contraindications (−11%), correct definition for a measles outbreak (−21%), and how to treat a child with a severe adverse event following immunization (−10%). Twenty percent of providers reported cancelling ≥1 RI sessions during the SIA. Many RI process measures were at high proportions (>90%) before the SIA and remained high afterward, including proper vaccine administration techniques, proper vaccine waste management, and availability of vaccine carriers and vaccine registers.

**Conclusions:**

Focusing on activities that are easily linked between SIAs and RI services, such as using SIA high-risk village list to strengthen RI microplanning and examining ways to minimize the impact of an SIA on RI session scheduling, should be prioritized when implementing SIAs.

The Global Measles and Rubella Strategic Plan 2012–2020 outlines strategies for reaching elimination including 95% routine vaccination coverage for the first dose of measles-containing vaccine (MCV1), introduction of a second routine dose (MCV2) and use of supplementary immunization activities (SIAs) to provide an additional opportunity for measles vaccination, particularly for individuals in areas where routine immunization (RI) services are inadequate [1]. The Global Polio Eradication Initiative outlines a similar strategy of strengthening immunization systems while using mass polio vaccination campaigns where RI coverage is low. However, concern has long existed about the potentially negative impact that measles SIAs, polio campaigns, and similar vertical disease control, elimination, and eradication activities could have on the routine health system, including RI services [2–5].

The concern about negative effects of SIAs on RI relates in part to the similarities and differences between the 2 approaches. RI services seek to regularly provide all recommended vaccines in a country’s immunization schedule to a preset target age group (usually infants), through either health facilities or outreach sites, whereas SIAs usually provide 1 or 2 vaccines during a short time frame to a wider age range. However, SIAs and RI services have substantial overlap in resource requirements; specifically, SIA vaccinators are often the same healthcare providers who provide RI and other primary health services. Indeed, studies indicate SIAs can negatively affect routine health service delivery temporarily owing to this human resource overlap [6–12].

These negative impacts need to be weighed against the potential benefits that SIAs can have on RI service delivery, including (1) more efficient use of overlapping resources, (2) the ability of well-conducted SIAs to reach underserved children previously unreached by RI services, and (3) consolidation of SIA and RI microplanning activities, which includes identification of hard-to-reach populations for determining where to place vaccination sites via catchment area maps or village line listings in the microplan. Program components related to vaccine administration, including definitions of adverse events, vaccine contraindications and other vaccine safety related topics, are generally similar across SIAs and RI services [13, 14].

In recognizing the importance of RI services in achieving measles and rubella goals and the need to manage any negative effects of SIAs, the Global Measles and Rubella Strategic Plan and the 2010 World Health Organization African region measles SIA guidelines called for using measles elimination activities to strengthen RI services [1, 13]. However, little published evidence is available illustrating how to use SIAs to strengthen RI services and what effects are incurred in doing so. During 2010–2013, global partners engaged in research activities designed to fill this knowledge gap [6, 15–17] and multiple interventions were proposed for evaluation. The interventions could occur during SIA training (eg, remind providers that measles vaccine safety also applies to routine vaccinations), SIA planning (eg, incorporate SIA tools such as maps and line listings of hard-to-reach communities into RI planning tools and use SIA to sensitize community members about the availability and benefits of RI services) and SIA preparation (eg, repair vaccine cold-chain equipment at health facilities and stock with a sufficient supply of vaccination registers, health cards, and tally sheets).

The 2012 Nepal measles, rubella, and polio SIA provided an opportunity to use an SIA to strengthen RI services. We developed an intervention package targeted at strengthening specific RI service components and viewed as feasible for integration into the SIA. We then assessed changes in RI process measures directly related to our intervention package and implementation of the SIA.

## MATERIALS AND METHODS

### Intervention Package Design

Approximately 6 months before the start of the SIA, the study partners and SIA organizers developed an intervention package designed to use the SIA for strengthening RI processes (Table 1). The team focused on 5 RI service components based either on those suggested previously in the literature or on previous efforts to use polio vaccination campaigns to strengthen RI services [13, 18, 19]: vaccine safety and administration, recording and reporting practices, supply chain, communications, and disease surveillance. The SIA vaccinator training and preparation activities already covered each of these topics to some extent, so the team believed that building on the existing SIA training areas could make this package feasible to implement.

Materials used within SIA training, social mobilization, supervision, and monitoring were modified with RI messaging [13] (Table 2). These RI messages included the routine immunization schedule; messages that providers could use with parents, such as where to go for routine vaccination and what parents should do about any observed adverse events following immunization (AEFIs); messages that providers should give parents during RI sessions; reminders about correct contraindications for measles vaccination; and prompts about how various vaccine administration techniques learned in the SIA were also applicable to RI.

New materials and new activities were also introduced. For instance, a new RI invitation card was created to be provided to parents of children eligible for routine vaccinations when they came for the SIA. Providers were taught to ensure that the hard-to-reach villages they identified for the SIA were incorporated into the RI microplan to ensure these areas were covered with outreach sessions. The intervention package was deployed nationwide during SIA preparation and during and after implementation, as applicable; the SIA in the Central Region, where our evaluation occurred, was between 14 December 2012 and 14 January 2013. The entire SIA targeted 5.7 million children 9 months to 14 years of age and reached 91% of these children.

### Evaluation Design

Although the intervention package was deployed throughout the country, we selected only 1 of Nepal’s 5 development regions, the Central Region, for the evaluation, owing to limited study funding. We used a pre- and postintervention assessment design, with the pre-SIA health facility-based survey in January 2012 and the post-SIA survey in September 2013.

Each development region in Nepal is composed of 3 ecological zones: mountains, hills, and plains. We stratified our health facility sampling frame by ecological zone to obtain a representative sample by zone. Generally, each village development committee (VDC) has only 1 public health facility of interest (ie, sub health post, health post or primary health care center), so we used a sampling frame of VDCs with the assumption that 1 VDC was equivalent to 1 health facility. Using simple random sampling, VDCs within each zone were sampled based on proportional allocation to each ecological zone; however, VDCs in the mountains zone were oversampled to lower the estimated margin of error. Study funds were available to sample 100 health facilities of the 1219 in the Central Region. Based on this sample, the study was powered to detect a 9% change in a given evaluation indicator with an initial response distribution of 50%, using an α level of .05 and a matched analysis with a McNemar test.

The pre- and post-SIA surveys included (1) provider knowledge, attitudes, and practices questionnaire and (2) observations of a single day of RI sessions per facility. The same health facilities were visited in both pre- and post-SIA surveys to measure any change in the indicator measurement.

### Evaluation Indicators and Data Collection

The pre- and post-SIA surveys included questions aimed at assessing changes in RI system process measures that could be influenced by our intervention package and by the SIA processes, including cold-chain equipment purchase and repair performed for the SIA. After a 2-day data collection training workshop, data collectors from a local agency (Center for Molecular Development, Nepal) with experience conducting household or health facility–based surveys were paired in teams to travel to assigned locations. Data collectors were selected who had experience conducting surveys and had a health education background.

At each selected health facility, the provider who administered the majority of the routine vaccinations was interviewed using the structured health facility questionnaire. After administering the questionnaire, the data collector observed and documented the provider vaccinating for the remainder of the daily routine vaccination session. Observations were collected on the facility questionnaire and included which vaccines were given to each child, the method of vaccine injection, waste disposal techniques, documentation of each vaccination administered, and messages provided to the child’s caregiver. Each interview with a provider took about 30 minutes, and observations generally lasted 4 hours. A refresher workshop for data collectors occurred before the post-SIA survey; 20 of 24 pre-SIA survey data collectors also conducted the post-SIA survey.

### Data Analysis

All questions included in the pre-SIA survey were repeated in the post-SIA survey. To determine whether responses to measured indicators changed significantly between surveys for the same health facility, we used a McNemar test for binary variables and a paired *t* test for any continuous variables. We considered *P* values ≤.05 as a marker of statistically significant change in these latter tests.

## RESULTS

In total, 100 healthcare providers (1 per sampled health facility) were interviewed in the pre-SIA survey; all 100 pre-SIA survey health facilities were revisited in the post-SIA survey. All providers who were initially contacted agreed to take part in the survey, for a 100% response rate. In the post-SIA survey, 10 providers were new since the pre-SIA survey.

### Vaccine Safety and Administration

Before and after the SIA, nearly all providers were observed using proper vaccination technique, with no significant changes to the proportion of providers who did not touch the needle during injection (pre-SIA, 97%; post-SIA, 99%; *P* = .30) and provided a measles vaccine injection at a 45° angle (pre-SIA, 87%; post-SIA, 90%; *P* = .77) (Table 3). Syringe needles were observed to be sticking out of the safety box in 5% (*P* = .66) of facilities both before and after the SIA.

A minority of providers reported they would delay vaccination if a mother were reluctant to have a mildly ill child receive vaccination, but this proportion significantly increased from 26% before the SIA to 37% after it (*P* = .047). In addition, the proportion of providers who indicated they would encourage the reluctant mother to vaccinate the mildly ill child immediately decreased significantly after compared with before the SIA (pre-SIA: 85%; post-SIA, 65%; *P* < .001)

The majority of surveyed providers could name ≥1 possible adverse event from measles vaccination both before and after the SIA, and improvements were seen in knowledge about AEFIs (Table 3). After the SIA, the proportion of providers who knew that pain and tenderness at the injection site is an AEFI increased significantly by 11% (pre-SIA, 19%; post-SIA, 32%; *P* = .02), and the proportion who knew that local swelling is an AFEI increased from 40% to 46% (*P* = .40). The proportion who could name fever as an AEFI decreased nonsignificantly, from 74% to 65% (*P* = .18). No significant change occurred in the proportion of providers observed asking parents to wait at the health center for a short period after vaccination for AEFI monitoring or informing parents about potential AEFIs (pre-SIA, 7%; post-SIA, 10%; *P* = .32). After the SIA, the proportion of providers who indicated they would treat a child with a severe AEFI decreased significantly from 94% to 83% (*P* = .02), and the proportion who stated they were uncertain how to respond increased significantly from 4% to 14% (*P* = .02).

### RI Planning

After the SIA, a significantly higher proportion of providers had an RI microplan than before the SIA (pre-SIA: 20%; post-SIA, 37%; *P* = .049) and a map of the catchment area (pre-SIA, 20%; post-SIA, 32%; *P* = .04) SIA (Table 4). In comparison, after the SIA, 57% of providers (95% confidence interval [CI], 48%–66%) had a measles SIA microplan. For the SIA, 42% of providers (95% CI, 32%–52%) reported developing a list of high-risk communities; of those, 62% (48%–77%) used this list to identify new RI outreach sites. After the SIA, a significantly higher percentage of providers reported holding all scheduled outreach vaccination sessions (pre-SIA, 83%; post-SIA, 96%; *P* = .008). However, during the SIA, 20% (95% CI, 13%–28%) of providers reported cancelling routine vaccination sessions owing to the SIA. Finally, a lower proportion of respondents provided an accurate definition of a suspected measles outbreak (ie, >5 suspected measles cases in VDC in 1 month) after the SIA than before it (pre-SIA, 95%; post-SIA, 74%; *P* < .001), and a higher proportion were uncertain of the criteria (pre-SIA, 5%; post-SIA, 16%; *P* = .02).

### RI Delivery

After the SIA, compared with before it, a significantly higher proportion (17%) of providers were observed to always tell parents when to bring the child for the next routine vaccination (4%; *P* = .04) (Table 4). The proportion who sometimes provided this reminder changed little over time (pre-SIA, 70%; post-SIA, 66%; *P* = .59), but the proportion who never provided it decreased significantly over time (pre-SIA, 26%; post-SIA, 18%; *P* = .048)

Nearly all providers had an immunization register before (100%) and after (96%; *P* = .07) the SIA (Table 4). In comparison, before the SIA, only 51% had RI tally sheets; this increased significantly to 66% (*P* = .02) after the SIA. Both before and after the SIA, approximately 60% of providers (*P* = .74) were observed always documenting vaccinations on the register, and approximately 35% were observed sometimes doing so. The proportion of providers who always documented the vaccination on the child’s health card increased from 43% before the SIA to 53% after it (*P* = .06).

### Vaccine Cold Chain

Nearly all health facilities had a usable vaccine carrier before (98%) and after (92%; *P* = .07) the SIA, with no significant change over time (Table 4). The proportion of facilities with a refrigerator to store vaccine significantly increased by 9% after the SIA, although the post-SIA proportion was still low (post-SIA, 23%; *P* = .02). After the SIA, a significantly higher proportion of providers (81%) correctly knew that a reconstituted measles vaccine could be used for only 6 hours, compared with the pre-SIA proportion (67%; *P* = .01).

## DISCUSSION

This study is among the first to evaluate an intervention package designed to use an SIA to strengthen RI services and to assess effects of the SIA on RI process measures. In our study, we documented a variable impact of the SIA on these process measures. A number of RI system strengths before the SIA remained strong after the SIA, including most providers administering vaccines using safe, correct techniques, most able to name ≥1 common AEFI, and most facilities with a usable vaccine carrier. We also identified positive changes in specific vaccine safety and RI planning measures, and a substantial proportion of providers reported using SIA planning data to increase RI outreach delivery activities. However, certain RI operational indicators were weaker after the SIA, including provider knowledge about measles outbreak criteria and how to manage a severe AEFI, and belief in false contraindications for vaccination. These latter issues could be addressed through improved training. In addition, a substantial minority of providers reported cancelling RI sessions during the SIA. Most of these negative aspects can be overcome by adequate training of health providers and preplanning to secure additional resources, if necessary, to maintain functioning RI services.

The majority of the evidence for the impact of mass vaccination campaigns, including both polio and measles-rubella vaccination campaigns, on the routine delivery of vaccinations and other health services are based on qualitative research, generally using self-reports and retrospective record reviews [6, 20–22]. These studies documented qualitative improvements to the health system from disease elimination activities, including better staff skills from training opportunities during SIAs and stronger supply chains and health information systems.

In Nepal, we were able to use a quantitative approach to identify slight improvements to the vaccine cold chain and improvements to certain areas of vaccine knowledge. However, we also identified decreases in immunization-related knowledge among providers after the SIA. Many of these areas of knowledge related to policy definitions, such as proper contraindications for vaccination, how to respond to a severe AEFI, and when to declare a measles outbreak. These latter definitions are routinely covered in SIA trainings; however, inadequate implementation of SIA training (which was implemented as a cascade-style design from regional to facility level) could have led to increased uncertainty about these topic definitions among surveyed providers. Ensuring providers have an opportunity to ask questions during the SIA training, conducting supervision during cascade-style trainings to ensure that topics are not diluted at each step, and conducting knowledge checks with providers throughout the training may help resolve this issue.

In Nepal, we also observed that a substantial number of evaluated RI process measures were at high levels before the start of the SIA with very little room for improvement. After the SIA, most of these measures did not significantly improve but did remain at high levels. In retrospect, such results may not be surprising, because RI coverage levels for DTP3 and measles in Nepal (including the Central Region) have been near 90% since 2011, indicating a fairly robust RI system [23].

In a country with high RI coverage, there may be limited potential to demonstrate improvement in RI process measures. In this case, the goal would be to use the SIA to maintain high RI performance by ensuring that providers understand the applicability of information received during SIA training, checking and repairing cold-chain equipment, and managing potential disruption to RI sessions from the SIA. We did find that 20% of providers reported disruption to RI service delivery during the SIA, despite instructions during the SIA trainings to continue scheduling all routine vaccination sessions during the SIA period. Interruption of routine services during SIAs has been reported by several other studies, and solutions may require scheduling additional RI sessions immediately after the SIA to provide any missed vaccinations.

A few studies have quantitatively examined the effect of campaigns on routine vaccination coverage. These studies have largely reported negative or inconclusive effects of the campaigns on routine vaccination coverage, including studies of national polio immunization days in the Western Pacific and measles SIAs in South Africa [9, 10, 24]. A recently completed study from Cameroon assessing the effect of any type of health campaign on routine delivery of health services, including vaccinations, indicated significant negative effects, particularly where the frequency and intensity of campaigns is higher [12]. One often-mentioned difference between measles SIAs and polio campaigns is the need for a provider skilled in delivering an injectable vaccine during the measles SIA compared with the polio campaign where, depending on country practices, the orally delivered vaccine may be administered by a broader cadre of workers [7]. The skilled providers are often the ones who also manage and deliver routine vaccinations, so disruptions in staffing may be more common with a measles SIA.

During our post-SIA debriefings with national immunization program staff, we documented multiple lessons learned, which were later incorporated into global guidelines [15, 25]. During SIA planning, program staff expressed concern about focusing on RI system strengthening for fear of diluting SIA quality. These concerns resulted in focusing on interventions that could be incorporated into existing SIA activities, use existing SIA outputs, and not cause confusion among healthcare providers and target audiences. Although program staff agreed to the intervention package, in the debriefing they reported that assigning an RI focal point person or “champion” to ensure that the package would be emphasized throughout SIA planning and implementation may have resulted in better outcomes; staff believed that implementing the package was a distant second priority to the primary work of SIA implementation. In addition, although the intervention package was structured with feasible activities, these additional interventions may have still been too ambitious.

Given the acknowledged lack of strong support from program staff during implementation, it may have been better to limit resource intensive activities (eg, creating new materials, such as RI invitation cards or provider RI reference sheets) and rather focus on revising existing SIA training materials (eg, including messaging about the applicability of the covered topics on RI services) and on ensuring that RI vaccination sessions continue throughout the SIA period. Based on our evaluation findings, potential changes to interventions that formed our package include adding information to the vaccinator field resource (eg, how to identify and manage AEFIs) and incorporating a question- and-answer session at the end of the SIA vaccinator training to review key points, such as vaccine safety topics, measles outbreak definition, and RI messages to parents.

Our study has a number of limitations. First, we did not have a control group, because the SIA and intervention package were used throughout Nepal. However, during the time between the pre-SIA and post-SIA surveys, no other RI-strengthening interventions were conducted. Second, there was 10% turnover in providers from the pre-SIA to the post-SIA survey; however, in a subanalysis comparing providers who did not turn over with those who did, the overall findings did not change. This may be due, in part, to the many measured RI process measures that were facility specific rather than provider specific, such as vaccine cold-chain condition and RI microplan and map availability. Third, our direct observations of routine vaccination sessions were conducted on a single day, so practices may not have been representative of practices during other vaccination sessions.

Concerns about using a horizontal versus vertical approach to disease control and elimination are long-standing and are exemplified in the literature surrounding the use of SIAs versus routine service to deliver vaccinations [2]. A diagonal or oblique approach has been proposed, in which vertical activities, such as SIAs, are integrated across health system functions as part of a larger framework to strengthen the health system and thereby make full use of potential synergies between disease programs [26–29]. Although SIAs have generally not been actively used to strengthen RI services, they represent an opportunity to fulfill the diagonal approach.

In Nepal, we found that maximizing this opportunity was challenging, owing to the stand-alone complexity of managing a high-quality SIA to ensure that it achieved the much broader objective of disease elimination among a segment of the population beyond the age range targeted by RI services in Nepal. Widespread support for use of the SIA may require ensuring that the SIA planning committee assigns an RI-strengthening focal point person who supports and advocates for these RI-strengthening activities and any required funding. In addition, focusing on a limited number of RI service components for strengthening or maintenance during an SIA may help increase the feasibility of using an SIA to strengthen RI services. As countries work toward achieving disease elimination and control goals, SIAs will continue to play a critical role in reducing immunity gaps. Additional work is needed to find the most effective ways to maximize the positive impacts and minimize the negative impacts of these mass campaign interventions on delivery of RI services.

## Figures and Tables

**Table 1:** Modified and New Materials Developed for Intervention Package to Strengthen RI Services Using the 2012 Nepal Measles, Rubella, and Polio SIA

Material	New or Modified Tool	Description of Change	How/When Used	Target Audience
SIA guidelines	Modified	Addition of RI messages throughout guidelines where appropriate and addition of a new section on how to sustain the gains of the MR SIA through strengthening various RI activities	For development of the training materials	National, regional, and district health officials
SIA poster	Modified	Includes messages on importance of also having a child who is fully vaccinated with all nationally recommended vaccines and information on where the child can receive these vaccinations and recommended ages of vaccination	Posted within community, health facility, market areas 104 wk before SIA	SIA target population
RI invitation card	New	Card given when mother comes to booth to remind her to bring child back for RI services and where/when these services can be received	Distributed to mothers of infants when they come to an SIA booth	Caregiver of infants (age <2 y)
Vaccinator field resource	New	Key SIA operational and RI messages for vaccinators (4-fold brochure)	Distributed to vaccinator at SIA training and used during SIA	Vaccinator
FCHV field resource	New	Key SIA operational and RI messages for FCHVs (2- or 3-fold brochure)	Distributed to vaccinator at SIA training and used during SIA	FCHV
Training flipchart or presentation	Modified	Includes RI messages (see Table 3) on key slides within flipchart	Used during SIA training by district officials	District health official, vaccinator, FCHV
Supervision checklist	Modified	Checklist includes checking if RI messages are given and if providers have brochures	Used during SIA by supervisors	Supervisor
Rapid convenience survey	Modified	Survey includes checking whether the child is fully up to date on RI schedule and has RI vaccination card	Used during SIA by monitors	SIA monitor

Abbreviations: FCHV, female community health volunteer; RI, routine immunization; SIA, supplementary immunization activity.

**Table 2:** Newly Introduced or Modified Key Activities in Intervention Package

Material	Description of Change	How/When Used
Monitoring implementation of vaccinator/FCHV training	Supervisors are present during SIA trainings to help ensure effective implementation and provide an additional technical resource to assist with the training, if needed.	SIA training for vaccinators and FCHVs
Interactive training in the vaccinator and FCHV training sessions	Prior versions of vaccinator and FCHV measles SIA training relied primarily on didactic training methods; the goal of this intervention is to improve participant ability to learn information and skills more effectively through interactive training methods, such as discussion, demonstration, practice, and role play.	SIA training for vaccinators and FCHVs
Identification of hard-to-reach areas during SIA and updating of RI microplan	SIA emphasizes identifying hard-to-reach populations, and RI services must also do so; providers are to use the SIA form, where hard-to-reach populations will be recorded, including it in the RI microplan to ensure that outreach sessions specifically target these populations.	SIA preparation period and post-SIA microplan updating activity

Abbreviations: FCHV, female community health volunteer; RI, routine immunization; SIA, supplementary immunization activity.

**Table 3:** Changes in Vaccine Safety Indicators Based on Interviews With Healthcare Providers at 100 Health Facilities Before Versus After 2012 Nepal Measles, Rubella, and Polio SIA

Indicator (Provider Behavior or Response)	Proportion of Providers, %		*P* Value^a^
	Precampaign Survey	Postcampaign Survey	
Observed touching needle with finger during injection	3	1	.30
Observed not injecting measles vaccine at 45° angle	13	10	.77
Will inform supervisor if mother brings child with severe adverse reaction to vaccination	14	7	.09
Will provide treatment if mother brings child with severe adverse reaction to vaccination	94	83	.02
Does not know what to do if mother brings child with severe adverse reaction to vaccination	4	14	.02
Knows fever can happen after measles vaccination	74	65	.18
Knows pain and tenderness at site of injection can happen after measles vaccination	19	32	.02
Knows local swelling can happen after measles vaccination	40	46	.40
Knows measles vaccine can only be used for 6 h after reconstitution	67	81	.01

Abbreviation: SIA, supplementary immunization activity.

a *P* values from the McNemar test statistic comparing precampaign and postcampaign proportions.

**Table 4:** Changes in RI System Indicators at 100 Health Facilities Before Versus After 2012 Nepal Measles, Rubella, and Polio SIA

Indicator	Health Facilities, %		*P* Value^a^

	Precampaign Survey	Postcampaign Survey	
RI planning and delivery			
Immunization microplan available	20	38	.049
Available microplan includes hard-to-reach areas and populations	100	89	.04
Facility catchment map available	20	32	.04
All scheduled outreach sessions held in past 6 mo	83	96	.008
Any routine vaccination sessions cancelled during vaccination campaign	NA	20	NA
			
RI recording and reporting			
RI tally sheets available	51	66	.02
RI vaccination register available	100	96	.07
Vaccinator correctly records each observed vaccination in register	64	62	.74
Vaccinator correctly records each observed vaccination in child’s health card	43	53	.06

Vaccine supply chain			
Vaccine carrier available	98	92	.07
Refrigerator available	14	23	.02

Communications			
Vaccinator observed always telling parents to come for next routine vaccination when another vaccination is due	4	17	.04
Vaccinator observed sometimes telling parents to come for next routine vaccination when another vaccination is due	60	66	.31
If child has a mild illness, vaccinator will still encourage mother to have child vaccinated against measles	85	65	<.001
If child has a mild illness, vaccinator will delay measles vaccination until child is healthy	26	37	.047

Abbreviations: NA, not applicable; RI, routine immunization.; SIA, supplementary immunization activity.

a *P* values from the McNemar test statistic comparing precampaign and postcampaign proportions.

## References

[1] World Health Organization (2012). Global Measles and Rubella Strategic Plan 2012–2020.

[2] Dietz V, Cutts F (1997). The use of mass campaigns in the expanded program on immunization: a review of reported advantages and disadvantages. Int J Health Serv.

[3] Taylor CE, Taylor ME, Cutts F (1998). Ethical dilemmas in polio eradication. Am J Public Health.

[4] Taylor CE, Cutts F, Taylor ME (1997). Ethical dilemmas in current planning for polio eradication. Am J Public Health.

[5] Sutter RW, Cochi SL (1997). Comment: ethical dilemmas in worldwide polio eradication programs. Am J Public Health.

[6] Hanvoravongchai P, Mounier-Jack S, Oliveira Cruz V (2011). Impact of measles elimination activities on immunization services and health systems: findings from six countries. J Infect Dis.

[7] Griffiths UK, Mounier-Jack S, Oliveira-Cruz V, Balabanova D, Hanvoravongchai P, Ongolo P (2011). How can measles eradication strengthen health care systems?. J Infect Dis.

[8] Griffiths U, Hanvoravongchai P, Oliveira-Cruz V, Mounier-Jack S, Balabanova D (2010). A toolkit for assessing the impacts of measles eradication activities on immunization services and health systems at country level.

[9] Verguet S, Jassat W, Bertram MY (2013). Supplementary immunization activities (SIAs) in South Africa: comprehensive economic evaluation of an integrated child health delivery platform. Glob Health Action.

[10] Verguet S, Jassat W, Bertram MY (2013). Impact of supplemental immunisation activity (SIA) campaigns on health systems: findings from South Africa. J Epidemiol Community Health.

[11] Schreuder B, Kostermans C (2001). Global health strategies versus local primary health care priorities–a case study of national immunisation days in Southern Africa. S Afr Med J.

[12] Mounier-Jack S, Edengue JM, Lagarde M, Baonga SF, Ongolo-Zogo P (2016). One year of campaigns in Cameroon: effects on routine health services. Health Policy Plan.

[13] (2010). Measles SIAs planning & implementation field guide.

[14] Hersh BS, Carr RM, Fitzner J (2003). Ensuring injection safety during measles immunization campaigns: more than auto-disable syringes and safety boxes. J Infect Dis.

[15] Fields R, Dabbagh A, Jain M, Sagar KS (2013). Moving forward with strengthening routine immunization delivery as part of measles and rubella elimination activities. Vaccine.

[16] Koehlmoos TP, Uddin J, Sarma H (2011). Impact of measles eradication activities on routine immunization services and health systems in Bangladesh. J Infect Dis.

[17] World Health Organization (2013). Planning and implementing high quality supplementary immunization activities for measles and rubella and other injectable vaccines.

[18] Steinglass R, Fields R (2000). Practical checklist to use PEI to strengthen the routine immunization program, in technical consultation on the global eradication of poliomyelitis.

[19] Sodha SV, Dietz V (2015). Strengthening routine immunization systems to improve global vaccination coverage. Br Med Bull.

[20] Loevinsohn B, Aylward B, Steinglass R, Ogden E, Goodman T, Melgaard B (2002). Impact of targeted programs on health systems: a case study of the polio eradication initiative. Am J Public Health.

[21] Closser S, Cox K, Parris TM (2014). The impact of polio eradication on routine immunization and primary health care: a mixed-methods study. J Infect Dis.

[22] Gashut H, Hall A, Ndumbe P, Wafula E, Wouters A (2009). GPEI external evaluation— international spread team.

[23] WHO/UNICEF estimates of national immunization coverage (WUENIC).

[24] Aylward RB, Bilous J, Tangermann RH (1997). Strengthening routine immunization services in the Western Pacific through the eradication of poliomyelitis. J Infect Dis.

[25] World Health Organization (2013). Planning and implementing high quality supplementary immunization activities for injectable vaccines using an example of measles and rubella vaccines.

[26] Atun R, de Jongh T, Secci F, Ohiri K, Adeyi O (2010). Integration of targeted health interventions into health systems: a conceptual framework for analysis. Health Policy Plan.

[27] Atun RA, Bennett S, Duran A (2008). When do vertical (stand-alone) programmes have a place in health systems?.

[28] Sepúlveda J, Bustreo F, Tapia R (2006). Improvement of child survival in Mexico: the diagonal approach. Lancet.

[29] Orenstein WA, Seib K (2016). Beyond vertical and horizontal programs: a diagonal approach to building national immunization programs through measles elimination. Expert Rev Vaccines.

